# Cell-Free DNA Profiling to Discover Mechanisms of Exceptional Response to Cabozantinib Plus Panitumumab in a Patient With Treatment Refractory Metastatic Colorectal Cancer

**DOI:** 10.3389/fonc.2018.00305

**Published:** 2018-08-28

**Authors:** Jingquan Jia, Michael A. Morse, Rebecca J. Nagy, Richard B. Lanman, John H. Strickler

**Affiliations:** ^1^Department of Medicine, Duke University Medical Center, Durham, NC, United States; ^2^Guardant Health, Inc., Redwood City, CA, United States

**Keywords:** *MET* amplification, metastatic colorectal cancer, cabozantinib, cell-free DNA, ctDNA

## Abstract

*MET* amplification is rare in treatment-naïve metastatic colorectal cancer (CRC) tumors, but can emerge as a mechanism of resistance to anti-EGFR therapies. Preclinical and clinical data suggest that patients with *MET* amplified tumors benefit from MET-targeted therapy. Cabozantinib is an inhibitor of multiple tyrosine kinases, included c-MET. Panitumumab is an inhibitor of EGFR. This report describes a patient with *KRAS, NRAS*, and *BRAF* wild-type metastatic CRC who experienced disease progression on all standard chemotherapy and anti-EGFR antibody therapy. The patient was enrolled in a clinical trial evaluating the combination of cabozantinib plus panitumumab. After only 6 weeks of treatment, the patient experienced a significant anti-tumor response. Although tumor tissue was negative for *MET* amplification, molecular profiling of cell-free DNA (cfDNA) revealed *MET* amplification. This case represents the first report showing the activity of cabozantinib in combination with panitumumab in a patient with metastatic CRC, and suggests that *MET* amplification in cfDNA may be a biomarker of response. A clinical trial targeting *MET* amplified metastatic CRC is currently underway.

## Background

The receptor tyrosine kinase c-MET (mesenchymal-epithelial transition factor), is implicated in tumorigenesis, proliferation, invasiveness, metastasis, and resistance to cancer treatment (1). Encoded by the *MET* proto-oncogene, c-MET is a disulfide-linked glycoprotein consisting of an extracellular α-subunit and a membrane spanning β-subunit (1). Hepatocyte growth factor (HGF) is the only known ligand for c-MET, and is predominantly secreted in a paracrine fashion by stromal cells. HGF binding induces c-MET receptor dimerization which in turn activates various downstream signaling pathways (2). HGF/c-MET signaling plays an essential role in diverse physiological processes such as embryonic development, epithelial branching morphogenesis and postnatal organ regeneration (3). Aberrant MET activation can occur via multiple mechanisms, including *MET* gene amplification (4).

*MET* gene amplification has been observed in multiple tumor types, including colorectal cancer (CRC) (5, 6), gastric cancer (7, 8), genitourinary cancers (9), head and neck cancer (10), non-small cell lung cancer (NSCLC) (11, 12), neuroblastoma (13), and ovarian cancer (14, 15). *MET* amplification is one of the key mechanisms mediating both primary (16) and acquired resistance (17) to epidermal growth factor receptor (EGFR) inhibition in patients with NSCLC. It has been shown that *MET* amplification leads to acquired resistance to EGFR tyrosine kinase inhibitors (TKI)s by persistent activation of ERBB3 signaling (18) and *MET* amplification can be detected with or without the presence of the *EGFR* T790M “gatekeeper” mutation (19). The prevalence of *MET* amplification is low (~3 %) in patients with untreated NSCLC, but increases to 5–22% in patients who develop acquired resistance to EGFR TKI therapy (17, 19, 20). The emergence of *MET* amplification under the selective pressure of anti-EGFR therapy supports the notion that *MET* amplification is a driver of acquired treatment resistance (21).

In patients with metastatic CRC, *MET* amplification is associated with resistance to anti-EGFR antibodies, including cetuximab and panitumumab. In mice engrafted with *MET* amplified CRC tumors, treatment with cetuximab is ineffective, suggesting that *MET* amplification may be responsible for intrinsic resistance to anti-EGFR antibodies (22). Functional crosstalk between c-MET and EGFR provides compensatory signal transduction leading to constitutive activation of downstream MAPK and PI3K pathways, thereby circumventing upstream EGFR blockade (23). *MET* amplification is found in less than 3% of patients with metastatic CRC who have not been exposed to anti-EGFR antibodies. Given the fitness advantage of *MET* amplification under the selective pressure of anti-EGFR therapies, *MET* amplification is much more common after exposure to anti-EGFR antibodies. Bardelli et al. (22) found that *MET* amplification emerged in post-treatment tumor biopsies of 3 out of 7 patients with metastatic CRC who developed acquired resistance to cetuximab or panitumumab (22). In a separate cohort of 22 patients with *RAS* and *BRAF* wild-type, HER2/MET negative metastatic CRC who developed resistance to anti-EGFR therapy, *in situ* hybridization (ISH) of the tumor tissue biopsies identified *MET* amplification as one of the most common genomic alterations (24).

Molecular profiling of blood-based circulating cell-free DNA (cfDNA) also supports *MET* amplification as a driver of EGFR antibody resistance. In a study by Siravegna et al. *MET* amplification was detected in 3 out of 16 patients who developed acquired resistance to anti-EGFR therapy (25). In another cohort of 53 patients with metastatic CRC, *MET* amplification was detected in in 22.6% (12/53) of patients with RAS wild-type tumors after exposure to anti-EGFR antibody therapy, but not found at an elevated frequency in anti-EGFR antibody-naïve patients (26). In addition, *MET* amplification was uncommon in *RAS* mutated patients (26). These findings have two major implications. First, it supports the utility of *MET* amplification as a biomarker of treatment resistance in patients with *RAS* wild-type EGFR antibody refractory metastatic CRC. Second, it demonstrates that *MET* amplification can be detected in cfDNA, thus supporting the clinical validity of cfDNA profiling to select patients for MET-targeted therapy.

The efficacy of MET inhibition in anti-EGFR antibody refractory metastatic CRC has been demonstrated in many preclinical studies. For example, in *MET* amplified patient-derived colorectal cancer xenograft models, MET tyrosine kinase inhibitors (TKIs) reversed resistance to EGFR blockade (22). Synergistic inhibitory effects between MET TKI and EGFR blockade was shown in a CRC xenograft mouse model expressing human HGF, where more pronounced tumor regression with concomitant MET TKI and cetuximab was observed *in vivo* in comparison to MET inhibition or cetuximab alone (27).

Cabozantinib is an orally bioavailable TKI that targets c-MET and VEGFR2, as well as RET, ROS1, AXL, KIT, and TIE-2. Cabozantinib is approved by the United States Food and Drug Administration (FDA) for use as monotherapy for metastatic medullary thyroid cancer^1^ and advanced renal cell carcinoma^2^. Panitumumab is an anti-EGFR monoclonal antibody FDA-approved for use in patients with *KRAS* and *NRAS* wild-type metastatic CRC^3^. Here we present a case report of a dramatic response to cabozantinib and panitumumab in a patient with *MET* amplified, EGFR antibody refractory metastatic CRC.

## Case report

**Figure 1:**
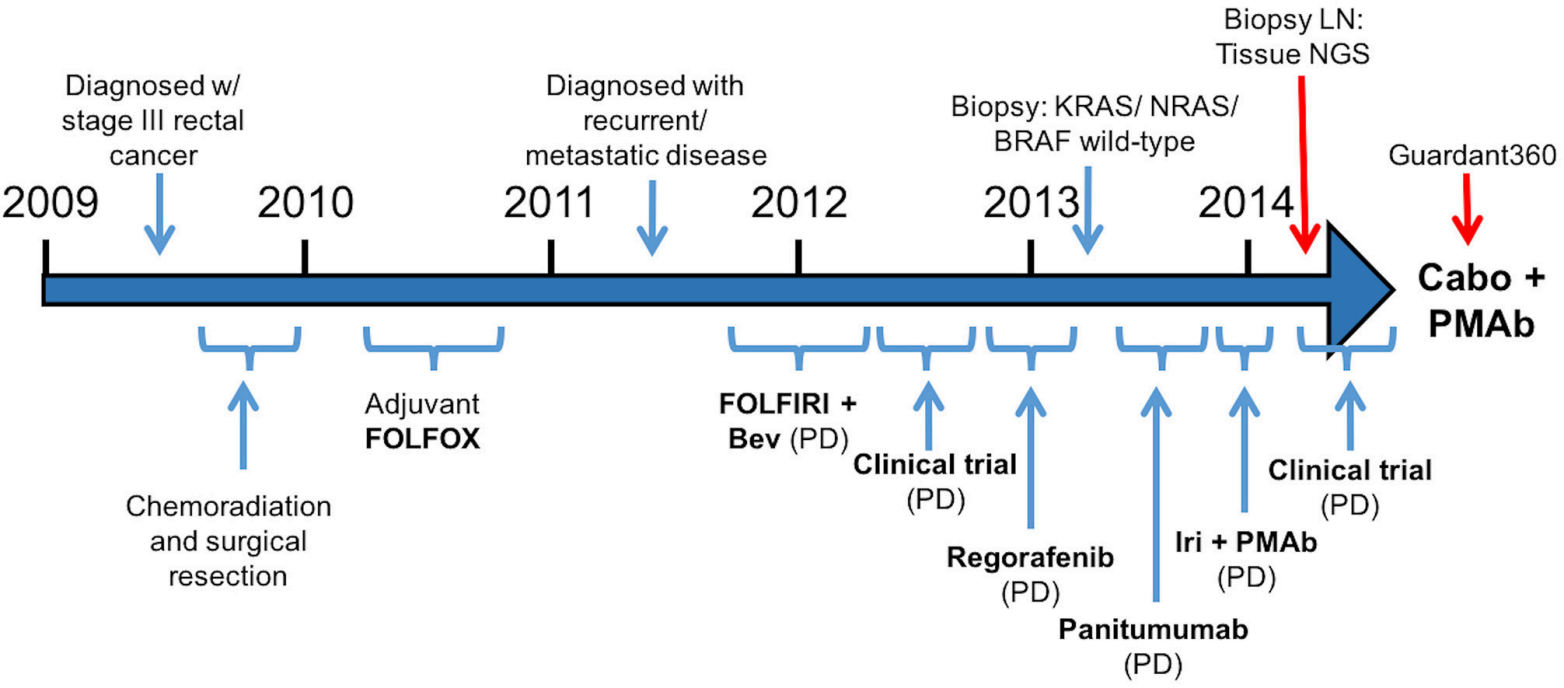
Treatment course (Bev, bevacizumab; Iri, irinotecan; PMAb, panitumumab; LN, lymph node; Tissue NGS, tissue next generation sequencing; Guardant360, cell free DNA profiling; Cabo, cabozantinib).

A 57-year-old male was initially diagnosed with locally advanced rectal cancer (T3N1M0) and treated with neoadjuvant chemoradiation followed by surgical resection (Figure 1). He subsequently received adjuvant modified (m) FOLFOX6 followed by colostomy reversal.

Two years later, CT imaging demonstrated new retroperitoneal lymphadenopathy suspicious for metastatic disease, and retroperitoneal lymph node (LN) biopsy revealed metastatic adenocarcinoma consistent with CRC primary. He received first-line treatment with FOLFIRI plus bevacizumab, but eventually experienced disease progression. He then progressed on a clinical trial combining capecitabine with an investigational therapy, followed by progression on regorafenib.

**Figure 2:**
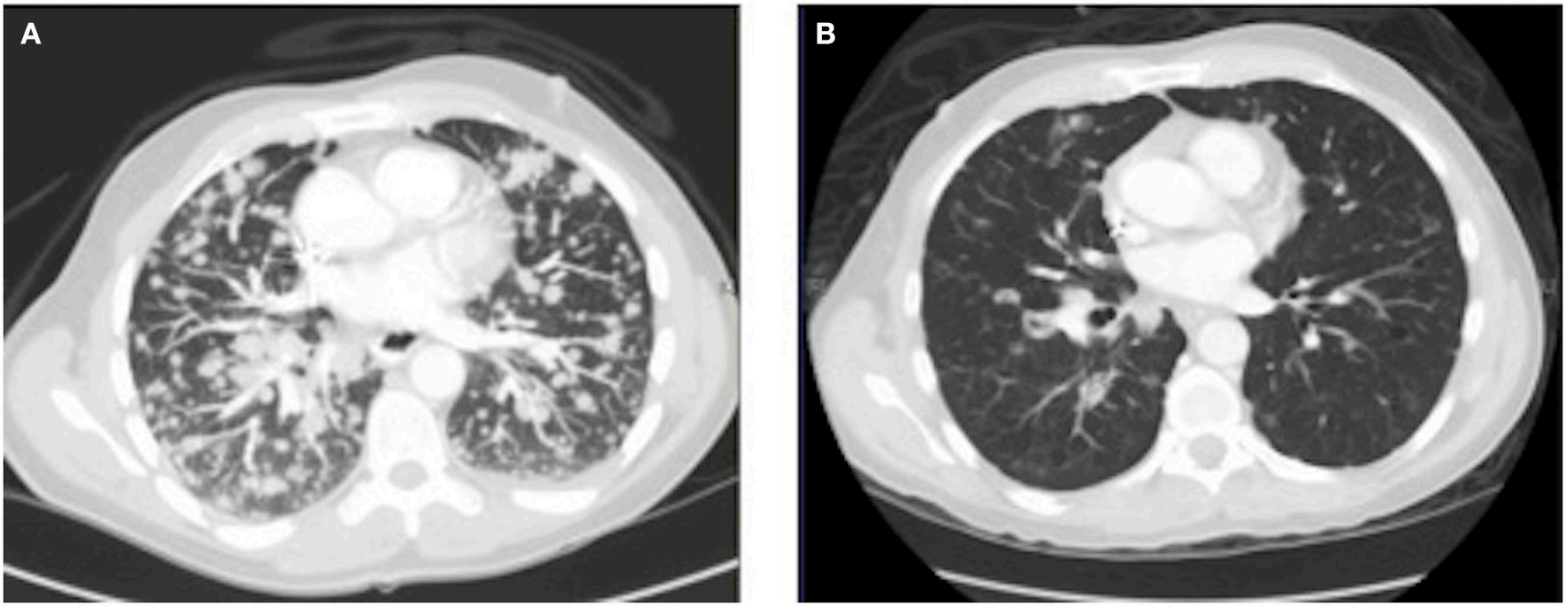
Chest CT image **(A)** before the start of cabozantinib plus panitumumab and **(B)** after 42 days of cabozantinib plus panitumumab.

**Figure 3:**
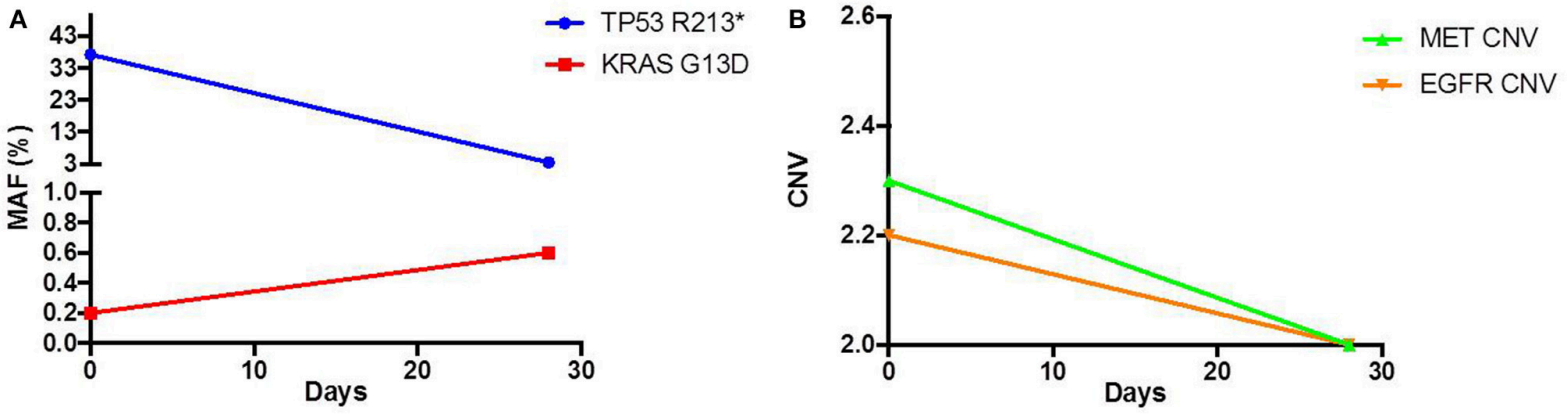
Pre and Post treatment cfDNA profile of **(A)** mutant allele frequency (MAF) and **(B)** copy number variation (CNV).

As his tumor was *KRAS* and *NRAS* wild-type, he was then treated with anti-EGFR antibody therapy (panitumumab). After 7 months of disease control, imaging revealed a new hypermetabolic LN at the right common iliac chain, and irinotecan was added to panitumumab. This treatment was eventually discontinued due to disease progression. A new biopsy of a mediastinal LN was performed and next generation sequencing (NGS) revealed that the tumor was still *KRAS, NRAS*, and *BRAF* wild-type, and there was no evidence of *MET* amplification (*see* Table 1). After progression on another phase I clinical trial with an investigational therapy, he was then enrolled in a phase Ib clinical trial combining cabozantinib and panitumumab (NCT02008383). At the time that he started treatment, he was increasingly symptomatic due to extensive pulmonary metastases, with worsening cough and shortness of breath. After ~6 weeks of treatment, CT demonstrated dramatic improvement in his pulmonary tumor burden (see Figure 2), as well as resolution of dyspnea and cough. As part of the trial protocol, plasma-EDTA was collected before the start of treatment to explore potential drivers of treatment response and/or resistance. CfDNA profiling utilizing a 54-gene targeted NGS panel (Guardant 360™) was performed on this sample. Blood-based profiling revealed subclonal *EGFR, KRAS* and *BRAF* resistance mutations. Additionally, *EGFR* amplification and *MET* amplification were observed in cfDNA, but not in tissue obtained 3 months prior (Table 1). Unfortunately his treatment course was complicated by anastomotic dehiscence and leak with abscess evolution. Because the dehiscence was apparently related to marked treatment response and tumor involution, treatment was discontinued. CfDNA profiling performed after 28 days of treatment revealed loss of *MET* and *EGFR* amplification (Figure 3A), while the mutant allele frequency (MAF) of *KRAS* G13D increased from 0.3 to 0.6%. There was also a nearly 10-fold decrease in the MAF of *TP53* R213^*^ post treatment, likely correlating with the dramatic reduction of tumor burden (Figure 3B).

**Table 1:** Tissue-based next-generation sequencing (NGS) and blood-based cfDNA NGS.

**Gene**	**LN biopsy (NGS) (2/27/2014)**	**Blood cfDNA (5/28/2014)**
*APC*	Y935fs^*^1	Y935N^†^
*BRAF*	Not detected	G466E^†^
*EGFR*	G465R - subclonal	G465R^†^, G465E^†^, S464L^†^
		Amplified (pCN 2.2)
*FAM123B*	G348fs^*^29	Not tested
*FGFR1*	Amplified	Not tested
*KRAS*	Not detected	G13D^†^, G12S^†^, Q61H^†^
*MET*	Not detected	Amplified (pCN 2.3)
*NF1*	Rearrangement int30	Not tested
*TP53*	R213^*^	R213^*^

† Minor alterations: Defined as alterations with relative variant allele frequency (rVAF) less than 10% of the alteration with the highest VAF. In this case TP53 R213

* *is the alteration with the highest VAF*.

After 2 months off therapy, his CEA increased and his dyspnea and cough returned. Capecitabine was initiated and panitumumab was added 2 months later for additional control. He experienced brief stabilization of disease on capecitabine and panitumumab, with subjective improvement of his pulmonary symptoms. He then experienced disease progression and was transitioned to hospice. He died ~10 months after discontinuing cabozantinib and panitumumab.

## Discussion

Despite advances in the treatment of CRC, it remains the second leading cause of cancer-related death in the United States (28). Patients with *RAS* wild-type metastatic CRC are eligible for treatment with the anti-EGFR antibodies panitumumab or cetuximab (29, 30). The clinical benefit of anti-EGFR antibodies is modest, with a single agent response rate of ~20% and a median progression free survival of 4 months (31). Even among patients who experience benefit from EGFR antibodies, acquired resistance is nearly universal (32, 33).

Multiple mechanisms of acquired resistance to anti-EGFR therapy have been identified in metastatic CRC, including *BRAF* mutations, acquired mutations in the *EGFR* extracellular domain (34, 35), *KRAS* and *NRAS* mutations (36, 37), and *MET* amplification (22) and these mutations often co-occur (38). Of these, *MET* amplification is potentially treatable with tyrosine kinase inhibitors and antibodies in development. Previous preclinical studies have demonstrated the potential activity of MET inhibitors in treating cetuximab or panitumumab refractory metastatic CRC. For example, treatment with a selective MET TKI successfully restored sensitivity to cetuximab in two cetuximab-resistant human colon cancer cell lines *in vitro*. The two cell lines displayed MET signaling pathway activation but *MET* amplification was not examined (39). Using a CRC cell-line harboring *MET* amplification in a murine xenograft model derived from a patient who developed acquired resistance to anti-EGFR therapy, tumor growth *in vivo* was effectively inhibited by crizotinib, a MET/ALK inhibitor (22).

To date, several MET TKIs have been developed with variable kinase selectivity against c-MET. Many of these are under different stages of clinical evaluation, either alone or in combination with other targeted therapy in patients with advanced solid tumors (40, 41). The MErCuRIC phase I/II clinical trial aims to assess the safety and efficacy of the combination of crizotinib and a MEK1/2 inhibitor, binemetinib, in patients with MET over-expressing, RAS-mutant or RAS wild-type metastatic CRC (42). Subgroup analysis from this study suggested potential benefit in patients with high c-MET expression (43).

Although the mechanisms of treatment response in this case are not fully known, the response to cabozantinib and panitumumab may be explained by the restoration of sensitivity to panitumumab or potentially synergy from dual MET and EGFR inhibition. Of note, other objective responses to small molecule MET inhibitors have been reported in patients with metastatic NSCLC and gastric cancer who had *MET* amplification detected by cfDNA profiling (44, 45). Alternatively, the anti-angiogenic properties of cabozantinib may have contributed to the overall response. To better understand whether treatment with cabozantinib alone drives response for patients with *MET* amplified metastatic CRC, this trial has been expanded to treat patients with *MET* amplified metastatic CRC with cabozantinib monotherapy.

*MET* amplification is not routinely tested in clinical practice due to its low prevalence and unproven actionability. Additionally, access to treatment-refractory tumor tissue and molecular heterogeneity complicates testing efforts (24, 46). Given these limitations, cfDNA profiling may be the optimal approach for detection of *MET* amplification in the treatment refractory setting (47). In our patient, *MET* amplification was not detected in a tissue biopsy sample but was detected in plasma cfDNA ~3 months later (Table 1). One explanation for the discrepancy between tissue and blood profiling results is that *MET* amplification represented a subclonal alteration that was not consistently present throughout the same lesion (intratumoral heterogeneity) or between different lesions throughout the body (intertumoral heterogeneity), as described previously (48, 49). This possibility is supported by the notion that mutations known to mediate acquired anti-EGFR resistance, e.g., *KRAS* and *BRAF* mutations, were seen in blood, but not the LN biopsy, suggesting temporal evolution from a common clonal origin. Alternatively, tumor cells harboring *MET* amplification may not have been present at a sufficiently high allele frequency to be detected by the tissue-based NGS assay.

This is the first case, to our knowledge, showing the activity of cabozantinib in combination with panitumumab in a patient with metastatic CRC. *MET* amplification, which is an established driver of EGFR antibody resistance, may have played a critical role in sensitizing this refractory tumor to the combination of an anti-MET TKI and anti-EGFR therapy. To further understand the drivers of sensitivity and resistance, studies are ongoing to evaluate the activity of cabozantinib treatment, either alone or in combination with panitumumab, in *MET* amplified metastatic CRC.

## Conclusions

*MET* amplification is an important driver of EGFR antibody resistance. Anti-MET therapy is active in patients with *MET* amplified tumors, and may be a clinically actionable target in patients with *MET* amplified metastatic CRC. Clinical investigations are underway to determine how best to target *MET* amplified metastatic CRC, and to determine whether targeting *MET* amplification has meaningful anti-tumor activity. Furthermore, cfDNA profiling is a promising diagnostic technology to detect genomic alterations in the treatment refractory setting. Prospective clinical trials utilizing cfDNA to identify and treat *MET* amplified metastatic CRC are ongoing.

Written informed consent has been obtained from the next of kin for the publication of this case report.

## Ethics statement

This study was carried out in accordance with the recommendations and approval of the Duke University Cancer Protocol Committee and the Duke University Institutional Review Board. All subjects gave written informed consent in accordance with the Declaration of Helsinki.

## Author contributions

JJ contributed to conception and design, analysis and interpretation of data, writing, review, and revision of the manuscript, and technical, material support. MM, RN, RL, and JS contributed to conception and design, analysis and interpretation of data, writing, review, and revision of the manuscript, and technical/material support.

### Conflict of interest statement

JS is a consultant/advisory board member for Amgen, received commercial research grant support from Exelixis and has a patent pending for the treatment of metastatic colorectal cell carcinoma using cabozantinib plus panitumumab. RL has ownership interest (including patents) in Guardant Health. RN has ownership interest (including patents) in Guardant Health. The remaining authors declare that the research was conducted in the absence of any commercial or financial relationships that could be construed as a potential conflict of interest.

## Footnotes

^1^COMETRIQ (cabozantinib) full prescribing information, revised 1/2018.

^2^CABOMETYX (cabozantinib) full prescribing information, revised: 12/2017.

^3^VECTIBIX (panitumumab) full prescribing information, revised 6/2017.
